# Sodium-glucose cotransporter 2 inhibitors and heart failure: the best timing for the right patient

**DOI:** 10.1007/s10741-021-10170-1

**Published:** 2021-10-16

**Authors:** Paolo Severino, Andrea D’Amato, Silvia Prosperi, Bettina Costi, Danilo Angotti, Lucia Ilaria Birtolo, Cristina Chimenti, Carlo Lavalle, Viviana Maestrini, Massimo Mancone, Francesco Fedele

**Affiliations:** Department of Clinical, Internal, Anesthesiology and Cardiovascular Sciences, Sapienza University of Rome, Viale del Policlinico, 155, 00161 Rome, Italy

**Keywords:** Sodium-glucose cotransporter 2 inhibitors, Heart failure, Cardiovascular disease, Type 2 diabetes mellitus, Hospitalization, Cardiovascular death

## Abstract

Sodium-glucose cotransporter 2 inhibitors (SGLT2i), initially born as anti-diabetic drugs, have shown many beneficial effects on the cardiovascular system, in particular against heart failure (HF). HF is a complex and multifaceted disease that requires a comprehensive approach. It should not be considered as a simplistic cardiac disease, but a systemic disease that leads to multisystemic organ failure and death. Exploiting their pleiotropic effects, SGLT2i are a very valid tool for HF treatment. Beyond the indication to reduce HF hospitalization and death risk, in patients with diabetes mellitus at high cardiovascular risk or with established cardiovascular event, SGLT2i administration reported beneficial effects regarding the wide spectrum of HF manifestations and stages, independently by diabetes mellitus presence. Recent evidence focuses on HF rehospitalization, cardiac and all-cause death reduction, as well as symptoms and quality of life improvement, in patients with chronic HF or with a recent HF decompensation episode. Given the recent finding about the SGLT2i usefulness in HF patients, further studies are needed to define the best administration timing to maximize the SGLT2i-derived beneficial effects.

## Introduction

Sodium-glucose cotransporter 2 inhibitors (SGLT2i), initially born as anti-diabetic drugs, have shown beneficial effects in heart failure (HF) treatment. SGLT2i administration has already been recommended by the European Society of Cardiology (ESC) 2016 Cardiovascular Disease (CVD) Prevention in Clinical Practice Guidelines [1] and then, reconfirmed by the 2019 ESC Guidelines on Diabetes, Pre-Diabetes, and Cardiovascular Diseases [2] (Table 1). In both cases, SGLT2i use was restricted to diabetic patients with established CVD or at high cardiovascular (CV) risk. The latest observations have shown further benefits deriving from the use of this drugs class, extending their administration regardless of type 2 diabetes mellitus (T2DM) presence and across the wide CV risk factors spectrum. Moreover, many benefits deriving from SGLT2i administration have been observed in HF patients. In fact, currently, Dapagliflozin and Empagliflozin are recommended for the treatment of symptomatic heart failure with reduced ejection fraction (HFrEF), independently by the presence of diabetes mellitus, to reduce HF-related hospitalization and CV death risk [3–6] (Table 1). SGLT2i are very versatile and suitable in reducing CVD and HF progression. A meta-analysis of the main studies pointed out that SGLT2i, combined with a proper therapy for HF, may significantly reduce HF hospitalization, CV, and all-cause mortality rate, as well as the progression of kidney disease and its related adverse events [7]. Considering the cardio-nephroprotective role, they may slow renal function worsening, providing further benefits on HF-related cardio-renal syndrome. New perspectives regarding the use of SGLT2i are opening, regardless T2DM presence. In this regard, SGLT2i may have protective effects in the HF early phases, when the disease is not clinically overt yet, but morphological or functional or biohumoral heart alterations are already present.

**Table 1:** Schematic and summary indications regarding SGLT2i administration

	**CLASS OF EVIDENCE**	**SOURCE**
**Empagliflozin, Canagliflozin, or Dapagliflozin are recommended in patients with T2DM and CVD, or at very high/high CV risk, to reduce CV events**	I A	*2019 ESC Guidelines on diabetes, pre-diabetes, and cardiovascular diseases developed in collaboration with the EASD* [2]
• Empagliflozin is recommended in patients with T2DM and CVD to reduce the risk of death	I A	*2019 ESC Guidelines on diabetes, pre-diabetes, and cardiovascular diseases developed in collaboration with the EASD* [2]
• SGLT2i (Empagliflozin, Canagliflozin, and Dapagliflozin) are recommended to reduce the HF hospitalization risk in patients with T2DM	I A	*2019 ESC Guidelines on diabetes, pre-diabetes, and cardiovascular diseases developed in collaboration with the EASD* [2]
**SGLT2i are included in the early medical treatment for HFrEF, together with ACEi/ARNI, β-blockers and MRA**		*Preview of the 2021 ESC/HFA heart failure guidelines*
**Dapagliflozin and Empagliflozin are recommended to reduce the risk of HF hospitalization and death in symptomatic patients with HFrEF**		*Heart Failure Association of the European Society of Cardiology update on sodium-glucose co-transporter 2 inhibitors in heart failure* [5]
**Dapagliflozin and Empagliflozin are effective and safe in improving CV and renal endpoints in patients with an eGFR > 20–25 L/min/1.73 m**^**2**^ **Dapagliflozin is benefical and safe also in patients with eGFR < 20 mL/min/1.73 m**^**2**^		*Clinical practice update on heart failure 2019: pharmacotherapy, procedures, devices and patient management. An expert consensus meeting report of the Heart Failure Association of the European Society of Cardiology* [34]*; Patient profiling in heart failure for tailoring medical therapy. A consensus document of the Heart Failure Association of the European Society of Cardiology* [35]
**Empagliflozin showed a significant risk reduction for the composite outcome of CV death or hospitalization due to HF in HFpEF patients**		*EMPEROR-Preserved trial* [99]
**SGLT2i are indicated in patients with HFrEF (LVEF < 40%), with a NYHA class between II and IV, in association with a guideline directed medical therapy and regardless of T2DM**		*2021 Update to the 2017 ACC Expert Consensus Decision Pathway for Optimization of Heart Failure Treatment: Answers to 10 Pivotal Issues About Heart Failure With Reduced Ejection Fraction: A Report of the American College of Cardiology Solution Set Oversight Committee* [6]

CV: Cardiovascular; CVD: Cardiovascular disease; T2DM: Type 2 diabetes mellitus; HF: Heart failure; HFrEF: Heart failure with reduced ejection fraction; HFpEF: Heart failure with preserved ejection fraction, LVEF: Left ventricular ejection fraction; NYHA: New York heart association; eGFR: Estimated glomerular filtration rate; ACEi: Angiotensin-converting enzyme inhibitors; ARNI: Angiotensin receptor-neprilysin inhibitor; MRA: Mineralocorticoid receptor antagonist; ESC: European society of cardiology; ACC: American college of cardiology; EASD: European association for the study of diabetes; HFA: Heart failure association; EMPEROR: Empagliflozin outcome trial in patients with chronic heart failure and a reduced ejection fraction

Given the recent findings about the SGLT2i usefulness, also in patients with HF, an important issue concerning the SGLT2i best timing administration is currently ongoing. The aim of this review is to discuss the latest findings regarding SGLT2i use, both in HF and in diabetic patients at risk to develop HF, to provide a better comprehension of the best administration timing, according to patient clinical profile.

## The pleiotropic effects of sodium-glucose cotransporter 2 inhibitors: molecular and pathophysiological insights

SGLT2i were approved in 2008 by U.S. Food and Drug Administration (FDA) as anti-diabetic drugs [8]. Type 2 diabetes mellitus (T2DM) and heart are extremely interconnected. T2DM is a major risk factor for HF. Diabetic patients are hospitalized, due to HF, approximately four times more, if compared to non-diabetics [9–13]. In patients with T2DM, the HF development risk is more than twice, compared to non-diabetic population [14]. T2DM not only causes macroangiopathy, but it is itself a diabetic cardiomyopathy’s cause [12, 15]. SGLT2i have shown beneficial effects on CV system, while showing an adequate safety profile. On the contrary, pharmacovigilance studies highlighted that other antidiabetic medications, such as Thiazolidinediones, sulfonylureas, dipeptidyl peptidase 4 (DPP4) inhibitors, and insulin may lead to an increased CV risk [16–24].

SGLTs are ubiquitous proteins and, for this reason, SGLT2i show metabolic and hemodynamic effects, which justify their efficacy in T2DM and HF treatment. They slow down harm mechanisms leading to left ventricular remodelling and pathophysiological mechanisms associated with HF development and progression. However, HF is a systemic and progressive disease, and many structural, functional, and biohumoral alterations may appear long time before the overt clinical syndrome [25].

SGLT is a family of sodium glucose cotransporters, and its better-known isoform are SGLT1 and SGLT2. SGLT2 is expressed nearly exclusively in the kidney, while SGLT1 is also present in the intestine and heart [26, 27]. In the kidney, SGLT2 is localized in the first proximal convoluted tubule segment (S1) and exploiting the energy of the Na^+^/K^+^ ATPase pump, it transports glucose against gradient, in the peritubular capillaries. It is responsible for the uptake of 90% of the reabsorbed glucose. The other 10% of glucose is then reabsorbed by SGLT1, which is localized in the following proximal convoluted tubules segments (S2 and S3) [28]. Interestingly, SGLT2 is located close to the Na^+^/H^+^ exchanger 3 (NHE3), the major responsible for filtered sodium reuptake, in the proximal tubule. The two transporters act together and, for this reason, SGLT2 can directly affect natriuresis [29]. In HF, the NHE3 activity is markedly increased, and it is believed to determine both diuretic and endogenous natriuretic peptides resistance [30, 31]. The myocardium sodium-hydrogen exchanger 1 (NHE 1) hyperactivity results in intracellular sodium and calcium overload [32]. The SGLT2i ability to inhibit NHE-1 entails calcium overload prevention [33]. SGLT2 inhibition causes glycosuria, if glucose blood levels are above 40–80 mg/dL, reducing hypoglycemia risk. The glycosuria-induced osmotic diuretic effect results in volume contraction. Urinary glucose excretion requires, at least, moderately preserved renal function, thus SGLT2i, in particular Dapagliflozin and Empagliflozin, may be administered efficacy and safely up to an estimated glomerular filtration rate (eGFR) of 20–25 ml/min/1.73 m^2^ [34, 35] (Table 1).

From the pathophysiological point of view, SGLT2i’s effect on arterial blood pressure is determined by preload and afterload reduction. Since diabetic patients have micro- and macrovascular disease, the increased arterial stiffness and afterload promote a vicious circle, through which, the hemodynamic stress, induced by hypertension and reduced arterial blood pressure (BP) variability, causes organ damage. In this context, SGLT2i may play a pleiotropic effect, through which they determine arterial BP reduction [36, 37]. Several studies demonstrated that SGLT2i may improve endothelial function and aortic stiffness indices, inducing vasodilatation through voltage-gated potassium (*K*_v_) channels, and protein kinase G activation [38–44]. Interestingly, SGLT2i show to selectively reduce interstitial volume with minimal change in vascular volume, whereas loop diuretics may cause a reduction in both interstitial and intravascular volume [45]. In this regard, interstitial space fluid accumulation is responsible for the main HF symptoms, leading to peripheral and pulmonary congestion [46, 47].

Kidney dysfunction is a condition that often affects HF patients [48]. During HF progression, kidney dysfunction is due to HF-related hemodynamic effects, in particular kidney hypoperfusion [49], increased venous congestion and diuretics high doses long-term use [50]. T2DM is frequently associated with nephropathy, characterized by increased intraglomerular pressure and albuminuria. This is due to the afferent renal vessels’ dilation. SGLT2 are able to restore the tubulo-glomerular feedback, bringing more sodium to the macula densa, reducing the afferent vessels vasodilation, the intraglomerular pressure, and the albuminuria of about 40%, slowing down the kidney damage [51]. Dapagliflozin did not cause electrolyte imbalances in a study comparing it to Gliclazide [52]. In particular, the levels of chloride, magnesium, and sulphate were found to be higher, as well as urinary citrate excretion. The latter is a mechanism that contributes to the SGLT2i’s nephroprotective action, as it could be consequent to the citric acid cycle metabolism, the main aerobic energy source for cells [52]. Jhund et al. demonstrated that the Dapagliflozin administration reduces the eGFR reduction rate, showing a similar effect in HFrEF diabetic and non-diabetic patients. Moreover, its efficacy is independent by kidney function at baseline, in terms of HF worsening and CV death risk prevention [53]. Actually, the ongoing Rationale and protocol of the Dapagliflozin And Prevention of Adverse outcomes in Chronic Kidney Disease (DAPA-CKD) randomized controlled trial is investigating the Dapaglifozin’s efficacy and safety in renal and CV events occurrence reduction, in a population affected by chronic kidney disease, from stage 2 to 4, regardless of T2DM presence [54].

Lowering uric acid level of about 10–15%, SGLT2i contributes to prevent harmful downstream effects, such as inflammation, oxidative stress, and renin angiotensin aldosterone system (RAAS) hyperactivation [55]. This marks another important difference between SGLT2i and other diuretics: the latter are responsible for RAAS hyperactivation, uric acid blood levels increase, electrolytes loss and, above all, metabolic disorders. In fact, SGLT2i do not increase uric blood levels [56] and, although they stimulate tubule-glomerular feedback and induce plasma volume depletion, they determine small variation of serum magnesium, calcium, potassium, and phosphate values and no effects on serum sodium [57]. Moreover, stimulating magnesium blood level increase, SGLT2i have an anti-arrhythmic and cardioprotective effect [58, 59].

Due to urinary glucose loss, SGLT2i promote a direct caloric loss, reducing the body mass index (BMI) and the glycated hemoglobin 1c (HbA1c), between 0.5% and 1.0% [60].

SGLT2i-related urinary volume increase may induce hemoconcentration, hematocrit, as well as serum albumin increase [56, 61]. SGLT2i can restore a better heart oxygenation through hematocrit and hemoglobin levels increase, stopping the vicious cardio-renal involvement, seen in HF [62].

At the metabolic level, SGLT-2i show important implications. In patients with advanced diabetes and HF, fatty acids, and glucose oxidation pathways may be impaired and insufficient. In this context, these drugs induce a metabolic shift in favor of ketone bodies, a much more effective energy source for heart and kidney [63]. In this regard, SGLT2i have been associated with the development of a syndrome-defined euglycemic ketoacidosis. By decreasing the blood glucose values through the glycosuric mechanism, SGLT2i reduce the production of insulin increasing glucagon synthesis, responsible for ketones production, through lipid oxidation [64].

**Fig. 1:**
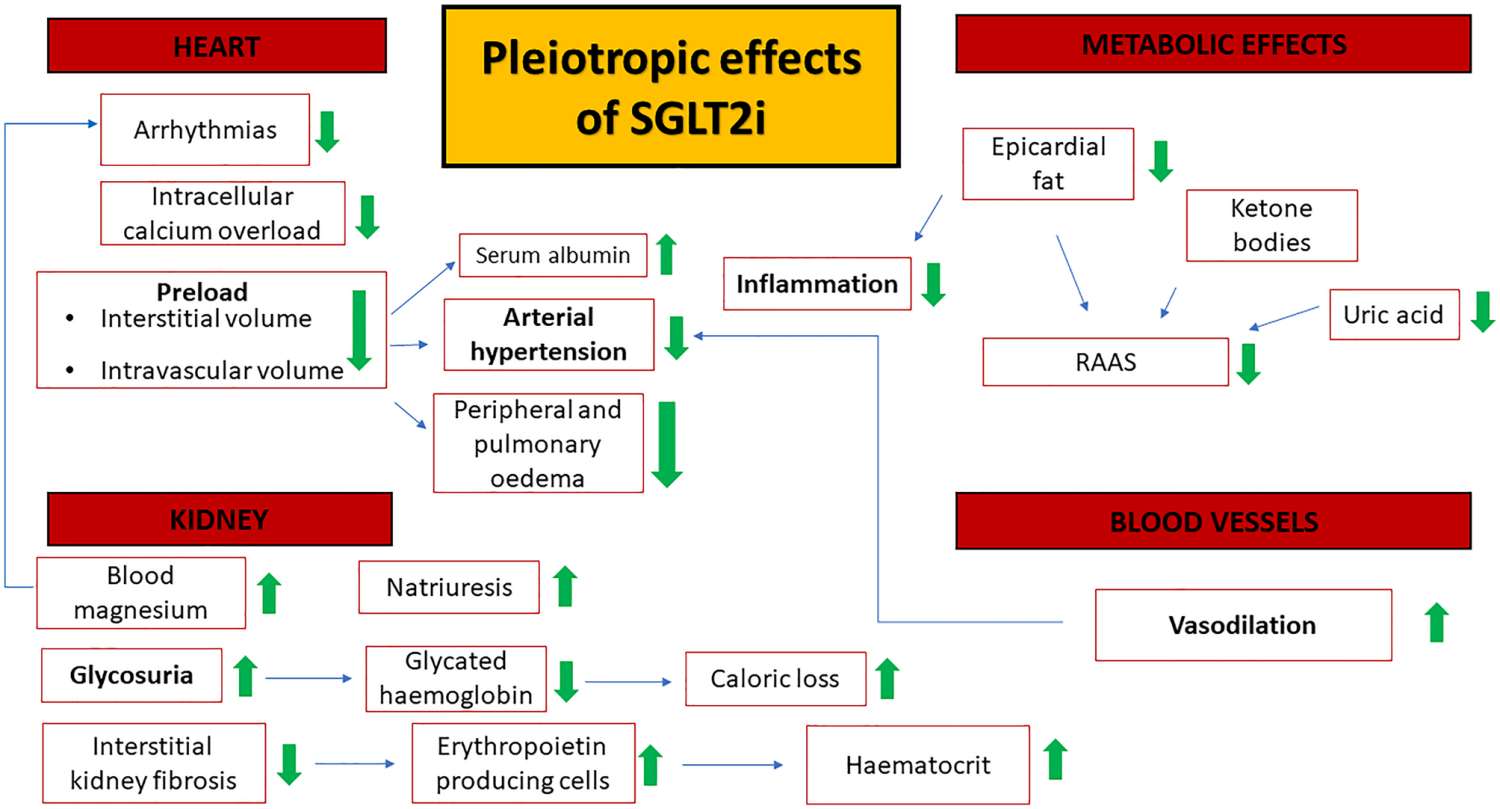
Sodium-glucose cotransporter 2 inhibitors (SGLT2i) pleiotropic effects. SGLT2i determine many multisystemic beneficial effects. They reduce volemia, through a natriuretic and glycosuric effect, reducing preload, as well as peripheral and pulmonary congestion. The arterial stiffness reduction promotes vasodilation and arterial pressure reduction. SGLT2i reduce metabolic imbalance and inflammatory response, observed in heart failure and type 2 diabetes mellitus. *SGLT2i*: sodium-glucose cotransporter2 inhibitors; *RAAS*: renin–angiotensin–aldosterone system

The main SGLT2i molecular and pathophysiological targets and effects are summarized in Fig. 1.

## Type 2 diabetes mellitus and heart failure: role of sodium-glucose cotransporter 2 inhibitors

Many studies and observations underline the effectiveness of SGLT2i use in patients with T2DM, without previous CV events and HF [5]. These observations opened the possibility to use SGLT2i as CV preventive drug, before HF onset, in patients with T2DM. In fact, in these patients, SGLT2i determine adjunctive benefits, reducing both the HF occurrence, as well as HF-related repeated hospitalizations and death. Empagliflozin, Dapagliflozin, Canagliflozin, and Ertugliflozin are actually recommended in patient with T2DM at high CV risk or with stable atherosclerotic cardiovascular disease (ASCVD), to reduce the hospitalization risk, due to HF [5] (Table 1). These observations have encouraged a meta-analysis on three important studies: CANagliflozin cardioVascular Assessment Study (CANVAS) [65], Empagliflozin Cardiovascular Outcome Event Trial in Type 2 Diabetes Mellitus Patients (EMPA-REG OUTCOME) [66] and Dapagliflozin Effect on Cardiovascular Events–Thrombolysis in Myocardial Infarction 58 (DECLARE-TIMI 58) [67], which confirmed that the Empagliflozin action is equally effective in patients with known HF and in those without [68]. In the CANVAS study, the primary outcome achievement was lower than in the EMPA-REG OUTCOME study and cardiovascular mortality reduction did not reach statistical significance. This could be explained by the different typology of patients enrolled in the two studies [69]. However, both studies show a notable reduction in hospitalization for HF, both in primary and secondary prevention, as long as it is conceivable that the use of SGLT2i in HF may be extended, also in absence of T2DM.

The Canagliflozin and Renal Events in Diabetes with Established Nephropathy Clinical Evaluation (CREDENCE) trial demonstrated the Canagliflozin’s efficacy in improving renal and CV outcome, in a population composed by patients with T2DM and chronic kidney disease, with or without CV disease. This study opened the possibility to use Canagliflozin to reduce the risk of major CV events and to improve renal function [70].

To reduce the occurrence of HF and other CV events, a strict and early control of CV risk factors is pivotal. In this regard, new evidences underline that SGLT2i act also on other CV risk factors, beyond T2DM, improving patient’s prognosis and reducing the risk of subsequent HF. Guidelines suggest the SGLT2i use in patients with T2DM with at least high CV risk or with ASCVD [1, 2]. In this regard, SGLT2i reduce arterial blood pressure, in patients with T2DM and hypertension, providing benefits in terms of CV risk prevention. It is well known that T2DM and arterial hypertension coexistence markedly worsens the ischemic heart disease, HF and cerebrovascular diseases risk. In fact, up to ¾ of diabetic complications may be due to arterial hypertension [36, 71]. For this reason, arterial blood pressure values control is crucial in diabetic patients, to reduce T2DM-related complications. In those patients, the lack of BP dipping during the night has been observed and it may be due to the increased volemia. Moreover, many diabetic hypertensive patients have masked and nocturnal hypertension, as well as excessive blood pressure rise during the morning, which associate with higher mortality rate [36, 72, 73]. SGLT2i hamper the hemodynamic stress, reducing arterial stiffness, circulating volume overload, and improving endothelial function [36, 74, 75]. Many studies report SGLT2i beneficial effects in the reduction of arterial stiffness and arterial BP profile improvement, shortly after therapy initiation, in diabetic patients [36, 38, 76, 77]. Moreover, Empagliflozin reduced arterial stiffness in younger patients with uncomplicated type 1 diabetes mellitus [36, 78]. SGLT2i contrast obstructive sleep apnea and CV complications, as demonstrated by Sawada et al., who showed apnoea-hypopnea index (AHI) reduction and glycated hemoglobin, as well as BMI improvement, in T2DM patients with obstructive sleep apnoea [36, 79].

As previously defined, in diabetic patients, SGLT2i provide benefits across the wide spectrum of different CV risk factors. This effect occurs both in patients with and without previous CV event. Fitchett et al. studied the Empagliflozin effect, varied by baseline CV risk, on cardiovascular outcome in the EMPA-REG OUTCOME trial [80]. In this context, Empagliflozin reduces HF hospitalization, CV and all-cause mortality, independently by baseline CV risk factors, as well as the presence of prior myocardial infarction and ischemic stroke, in T2DM patients with ASCVD. Moreover, the HF hospitalization reduction was observed both in patients with and without baseline HF. The CV outcomes reduction has been observed in both patients with and without coronary artery bypass grafting (CABG) baseline history [80–82] (Table 2). In patients with T2DM and ASCVD, the eValuation of ERTugliflozin effIcacy and Safety CardioVascular outcomes trial (VERTIS-CV) [83] confirmed that Ertugliflozin is a CV and renal safe and efficient drug.

**Table 2:** Main studies about SGLT2i according to type of patient and main cardiovascular findings

**TYPE OF PATIENT**	**STUDIES**	**MAIN FINDINGS**
**Patients with T2DM and high CV risk**	• CANagliflozin cardioVascular Assessment Study (**CANVAS**) • Empagliflozin Cardiovascular Outcome Event Trial in Type 2 Diabetes Mellitus Patients (**EMPA-REG OUTCOME**)	• Canagliflozin reduced CVD risk, both in primary and in secondary prevention • Empagliflozin reduced CV death, non-fatal MI or stroke and all-cause death
**Patients with T2DM and CVD**	• Dapagliflozin Effect on Cardiovascular Events–Thrombolysis in Myocardial Infarction 58 (**DECLARE-TIMI 58)** • EMPagliflozin comparative effectIvness and SafEty **(EMPRISE)**	• Dapagliflozin reduced MACE and composite CV death or HF hospitalization • Empagliflozin reduced MACE, HF-hospitalization ad all‐cause death
**Patients with HF:** • **HFrEF, regardless T2DM**	• Empagliflozin Outcome Trial in Patients with Chronic Heart Failure and a Reduced Ejection Fraction (**EMPEROR-Reduced)** trial • Dapagliflozin and Prevention of Adverse Outcomes in Heart Failure (**DAPA-HF**)	• Empagliflozin reduced CV death or HF hospitalization, total hospitalization, composite renal outcome and all-cause death • Dapagliflozin reduced CV death, HF hospitalizations and all-cause death
• **HFrEF and T2DM**	• Sotagliflozin on Cardiovascular Events in Patients With Type 2 Diabetes Post Worsening Heart Failure (**SOLOIST-WHF**)	• Sotagliflozin reduced CV death and hospitalization or urgent visit for HF

T2DM: type 2 diabetes mellitus; CV: Cardiovascular; CVD: Cardiovascular Disease; MI: Myocardial Infarction; MACE: Major Adverse Cardiac Events; HF: Heart Failure; HFrEF: Heart Failure with Reduced Ejection Fraction

The EMPagliflozin compaRative effectIveness and SafEty (EMPRISE) study demonstrates the HF hospitalization risk reduction in patients with T2DM treated with Empagliflozin, regardless of baseline CV disease presence [84] (Table 2). These results are consistent with EMPA-REG OUTCOME trial results, in terms of timing and importance [85]. The Comparative Effectiveness of Cardiovascular Outcomes in New Users of Sodium-Glucose Cotransporter-2 Inhibitors (CVD-REAL) study was a real-world study that evaluated the efficacy of SGLT2i in T2DM patients, compared to other glucose-lowering drugs, across several countries [86]. The 87% of patients did not show any known CV disease. The therapeutic management based on SGLT2i determined a reduction in relative risk of 51% for all-cause mortality, 39% for hospitalization due to HF and 46% for the composite of both, compared to other glucose-lowering drug therapeutic plan. Empagliflozin, Dapagliflozin, and Canagliflozin beneficial effect is mainly demonstrated in diabetic patients with high CV risk and regardless of HF, in terms of HF hospitalization prevention. Although SGLT2i effects are notably seen on HF, not all vascular events occurrence may be reduced through SGLT2i administration. In fact, Empagliflozin, Dapagliflozin, Canagliflozin, and Ertugliflozin have a neutral effect regarding stroke and myocardial infarction occurrence prevention [87, 88].

## Heart failure and sodium-glucose cotransporter 2 inhibitors: what is the best time of administration?

Despite SGLT2i have been initially used to treat T2DM, increasing evidence emphasize the effectiveness of SGLT2i use in patients with HF, regardless of T2DM presence. Dapagliflozin and Empagliflozin are recommended to reduce HF hospitalization and death in patients with HFrEF [5, 6] and this indication will be probably included in the next ESC HF guidelines (Table 1). HF is a clinical syndrome, as defined by The Universal Definition and Classification of Heart Failure Document [25]. It is characterized by symptoms and/or signs, determined by functional and/or structural cardiac abnormalities, and complemented by systemic or pulmonary congestion and high-circulating natriuretic peptides values [25]. A recently revised HF classification defines 4 stages of disease. Stage A defines patients with risk to develop HF, in which HF risk factors are present without any cardiac or biohumoral alterations. Stage B defines patients with a pre-HF condition, characterized by structural and/or functional cardiac abnormalities or biohumoral alterations, without symptoms. Stage C defines patients with actual or previous HF signs and/or symptoms, determined by a functional and/or structural cardiac alteration. Stage D defines the advanced HF that characterizes patients with severe HF symptoms and/or signs, with frequent recurrences and need of advanced therapies, such as left ventricular assist device, transplant or palliative care, despite an optimized medical therapy [25].

SGLT2i have been used efficiently in different HF stages, from the pre-HF to end-stage HF, as well as in the acute and chronic HF patients. In this context, in HF patients, SGLT2i have demonstrated to significantly reduce rehospitalization, all-cause death, and CV death. Moreover, they improve HF-related symptoms and life quality. This evidence is supported by pathophysiological considerations. In fact, it is simplistic to consider HF as a unique cardiac disease. HF is a multifaceted and complex syndrome characterized by a progressive involvement and dysfunction of systemic organs, such as lung, kidney, liver, brain, and bone marrow. HF could be defined as “the cancer of the heart” because HF management complexity derives from the progressive evolution of systemic dysfunction, leading progressively to multiorgan failure and finally to death. Therefore, as cancer is not treated according to presence of symptoms but to involvement of other organs, according to TNM classification, HF should be treated in order to avoid the progressive involvement and dysfunction of systemic organs, regardless of symptoms presence [89]. Exploiting the cardio-nephroprotective effects, SGLT2 may be administered in HF patients with systemic involvement, mainly characterized by kidney function worsening. A kidney injury, sustained by a low cardiac output, is often observed in HF patients’ clinical scenario, especially in case of HF acute exacerbation. In this case, SGLT2i administration may be considered after an initial patient stabilization, through inodilator and inotropes, if necessary, during the acute phase. However, SGLT2i may demonstrate beneficial effects also in HF patients without an overt systemic involvement, slowing down the systemic disease’s progression and contrasting heart and systemic alterations, that are already present in the early, pre-clinical phases of HF. Considering the SGLT2i pleiotropic and beneficial effects, what type of drug if not SGLT2i may efficiently counteract HF and its multifaceted pathophysiological pathways?

HF natural history is characterized by repeated hospitalizations. Hospitalization and death risks are particularly high during the HF vulnerable phase. This phase identifies the period corresponding to 6 months after an hospitalization due to acute HF [90, 91]. To reduce HF-related mortality and impact on health care system costs, it is important to reduce HF rehospitalization. Savarese et al. showed that the earlier Empagliflozin administration, after an index HF hospitalization, is associated with HF-related rehospitalization and the composite event of all-cause or CV death and HF rehospitalization reduction [90]. Early SGLT2i administration beneficial effect on HF-related adverse events reduction have been identified by the Empagliflozin Outcome Trial in Patients with Chronic Heart Failure and a Reduced Ejection Fraction (EMPEROR-Reduced) trial [92]. This trial demonstrated that the beneficial effect of Empagliflozin on HF hospitalization reduction, urgent and/or emergent HF visit, and death risk became significant starting from the twelfth day from the therapy begin and it is persistent during the treatment period [92] (Table 2). The Empagliflozin impact on HF hospitalization reduction has been demonstrated across several disease’s manifestation severity, inducing up to the 35% of intensive care need reduction. Moreover, the Empagliflozin effect has been observed also in stable and not decompensated HF patients. In this regard, those patients have higher possibility to experience a New York Heart Association (NYHA) class improvement, as well as reduced possibility to experience NYHA class worsening, compared to placebo. Patients treated with Empagliflozin do need less diuretic therapy intensification. Moreover, the SGLT2i effect has been observed also in patients already treated with angiotensin receptor neprilysin inhibitors (ARNI), mineralocorticoid receptor antagonists (MRA), and β-blockers. According to the Effect of Sotagliflozin on Cardiovascular Events in Patients With Type 2 Diabetes Post Worsening Heart Failure (SOLOIST-WHF) trial, the early Sotagliflozin administration, in patients with T2DM and recent HF worsening, was associated with a significant HF hospitalization, urgent visit and CV death reduction, compared to placebo [93] (Table 2). The early Sotagliflozin administration, shortly after, or before a discharge, after an episode of decompensated HF, represents an important opportunity to improve HF outcomes, in diabetic patients. Moreover, the Sotagliflozin beneficial effect have been demonstrated both in subgroups stratified according to left ventricular ejection fraction (LVEF) and the first Sotagliflozin dose administration timing.

In the Dapagliflozin and Prevention of Adverse Outcomes in Heart Failure (DAPA-HF) trial, the primary endpoint was a composite of HF exacerbation, defined as hospitalization for HF and/or urgent visit for intravenous therapy administration, and CV death. The population was made up of HFrEF patients, both with and without diabetes. Dapagliflozin was effective in reducing all individual endpoints and its effectiveness was irrespective of T2DM presence. Dapagliflozin administration improve symptoms and health status in patients with HF, as defined by the Kansas City Cardiomyopathy Questionnaire (KCCQ) [94] (Table 2).

Other aspects in the relationship between HF and SGLT2i have been investigated. In particular, their effectiveness according with disease duration, as well as the effect in HF outpatients and the combined effectiveness of SGLT2i with other drugs administered in HF patients. In this regard, Yeoh et al. analyzed the effect of Dapagliflozin in HF patients of DAPA-HF trial, according to HF duration [95]. In fact, HF is a progressive disease and patients with long-standing HF are older, with more comorbidities and more prone to develop the advanced disease with related complications. Dapagliflozin reduces in similar way the absolute risk of both long standing and recently diagnosed HFrEF patients. However, being the former at higher absolute risk, they reduce more the absolute risk in long standing HF patients, compared with recently diagnosed HFrEF patients. For this reason, Dapagliflozin represents an added value in a great population, the long-standing HF patients, who, having an optimized therapy, are considered not more susceptible of further clinical improvement, through medical treatment. Disease worsening represents a common and important event from a prognostic point of view, in HF outpatients. Docherty et al. conducted a prespecified analysis of DAPA-HF trial in HF outpatients [96]. They demonstrated a reduction of worsening risk in HF outpatients treated with Dapagliflozin, compared to placebo. Moreover, HF worsening, requiring oral therapy management, has the same prognostic meaning, compared to HF outpatients treated intravenously. In fact, disease worsening in HF outpatients leads to oral therapy assumption increase, which is associated with a threefold higher death risk. In these patients, both the HF worsening, requiring therapy management, and hospitalization events, are significantly reduced by Dapagliflozin administration.

Many pathophysiological pathways are involved in HF, and they determine the complexity of this syndrome. For this reason, an early multidrug regimen approach, based on β-blockers, MRA, Levosimendan, ARNI, and SGLT2i, may be beneficial, for HF treatment [97, 98]. Taking data from three important trials, Eplerenone in Mild Patients Hospitalization and Survival Study in Heart Failure (EMPHASIS-HF), Prospective Comparison of ARNI with ACEI (Angiotensin-Converting–Enzyme Inhibitor) to Determine Impact on Global Mortality and Morbidity in Heart Failure Trial (PARADIGM-HF) and DAPA-HF, Vaduganathan et al. evaluated the benefits of a comprehensive therapy, compared to conventional therapy, in chronic HF patients, in terms of overall and free adverse events survival [97]. They found that a comprehensive and early multidrug approach with MRA, beta-blocker, ARNI and SGLT2i gave further benefits, compared to conventional HF therapy (Table 1). In particular, reduction in hospital admission due to HF and CV death has been observed. Patients with a multidrug treatment for HF have demonstrated from 2.7 to 8.3 additional years, without HF hospitalization and CV events, and from 1.4 to 6.3 additional years of survival. Since a multidrug regimen approach for HF treatment can modify the course of HFrEF, the SGLT2i administration is successful and safe and it should be integrated early in HFrEF treatment, regardless of drugs administration order.

Finally, phase III of EMPEROR-preserved trial [99] showed that Empagliflozin significantly reduce the composite outcome of CV death or hospitalization due to HF, in patients with HF with preserved ejection fraction (HFpEF) (Table 1).

## Conclusions

HF is a complex disease that requires a comprehensive approach. Due to its multifaceted pathophysiology, HF should not be considered as a simplistic cardiac disease, but a systemic disease that progressively leads to multisystemic organ failure and death [89, 100–103]. The Universal Definition and Classification of Heart Failure Document includes in the HF definition also patients with risk factors alone and/or initial cardiac and biohumoral alterations, without an overt clinical syndrome [25]. Early identification and treatment of those patients represents a main target for cardiologists, in order to improve patients’ prognosis.

**Fig. 2:**
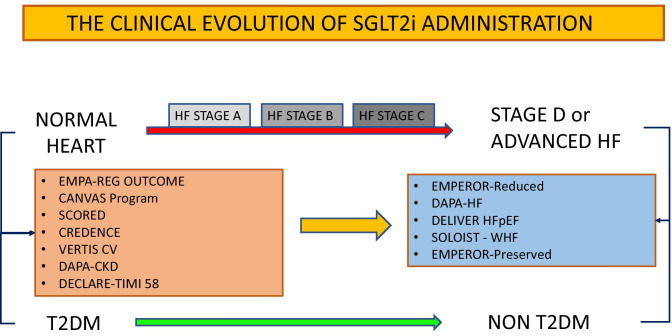
SGLT2i trials, diabetic status, and HF presence. The main trials regarding SGLT2i administration are summarized according to the HF presence and stage, as well as diabetic status of patients included. *SGLT2*: sodium-glucose cotransporter 2 inhibitors; *HF*: heart failure; *T2DM*: type 2 diabetes mellitus

The usefulness of SGLT2i regarding the wide spectrum of HF manifestations and stages have been demonstrated, independently by T2DM presence. Different SGLT2i trials are listed according to the diabetic status and HF presence of patients included (Fig. 2). Two main important points may be extrapolated by recent evidence about the SGLT2i administration. First, SGLT2i beneficial effects have been observed at different HF stages, both in acute and chronic HF patients. In particular, when SGLT2i were administered in patients with recent acute HF and decompensation episode, they demonstrated to significantly modify the disease’s progression, reducing HF rehospitalization, CV, and all-cause death. Secondly, SGLT2i significantly impact the prognosis of patients with T2DM and across the wide CV risk factors spectrum, in terms of HF prevention.

In conclusion, starting from the concept that, similarly to a cancer, HF should be treated in order to avoid the progressive involvement and dysfunction of systemic organs, regardless of symptoms presence, we are proposing that SGLT2i administration should be started as soon as possible, when clinical conditions allow it, regardless of HF hospitalization, mostly for their cardio- and nephroprotective effects. Moreover, their potential application may be already evaluated when morphological, functional or biohumoral heart abnormalities are already present, also in absence of overt HF clinical syndrome, thus not symptom-driven. Although actual SGLT2i indication regards patients with symptomatic chronic HFrEF, SGLT2i may represent a further treatment option to contrast left ventricular remodeling and the progressive systemic involvement seen in HF, in addition to first line therapy.

These observations are challenging the actual guidelines indication about the SGLT2i use, providing new perspectives for the HF patient management.
